# A grounded theory research protocol on an attempt to practice interprofessional collaborative care by a primary care clinic health professional fresh graduate in diabetes care

**DOI:** 10.3389/fmed.2023.1133948

**Published:** 2023-08-03

**Authors:** Kelly Sze Fang Num, Azimatun Noor Aizuddin, Seng Fah Tong, Mohd Shahrir Mohamed Said

**Affiliations:** ^1^Department of Public Health Medicine, Faculty of Medicine, Universiti Kebangsaan Malaysia, Kuala Lumpur, Malaysia; ^2^Division of Nutrition and Dietetics, School of Health Sciences, International Medical University, Kuala Lumpur, Malaysia; ^3^International Centre for Casemix and Clinical Coding, Hospital Canselor Tuanku Muhriz, Kuala Lumpur, Malaysia; ^4^Department of Family Medicine, Faculty of Medicine, Universiti Kebangsaan Malaysia, Kuala Lumpur, Malaysia; ^5^Department of Internal Medicine, Universiti Kebangsaan Malaysia Medical Center (UKMMC), Kuala Lumpur, Malaysia

**Keywords:** primary care clinics, diabetes care, fresh graduate health professional, interprofessional collaborative care, grounded theory protocol

## Abstract

Interprofessional collaborative care (IPCC) can improve the quality of care in patients with chronic diseases in primary care settings. In Malaysia, many medical and healthcare universities have adopted the concept of the interprofessional collaborative practice (IPCP) framework by the World Health Organization (WHO) and implemented interprofessional learning (IPL) in their curriculum to prepare fresh graduates for interprofessional collaboration (IPC) in the health workforce albeit in various degrees. However, there are potential challenges in putting what they have learned into practice, especially in managing chronic diseases due to the complexity of behavior changes required. Diabetes care is a classic example of such chronic disease management. This article presents a qualitative research protocol exploring the processes and challenges of fresh graduates attempting to practice IPC when managing diabetes mellitus (DM) in primary care clinics. A grounded theory (GT) approach will be adopted.

## 1. Introduction

Interprofessional collaborative care (IPCC) improves the quality of care and outcomes in patients with chronic diseases in primary care settings (1–7). Nevertheless, the extent of implementing IPC care practices varies and fresh graduates face great challenges. IPC is a complex process, and it requires a variety of actors to interact in social, cultural, and professional healthcare systems, especially sharing the decision-making process around health and social issues (8–10). It may be possible to overcome some of these challenges through better training of healthcare providers during their undergraduate years so that they are prepared for interprofessional collaborative practice (IPCP) (11).

When fresh graduates enter the workforce, they face several challenges in adapting and practicing what had been learned and experienced in university from interprofessional learning (IPL) (12–14). The real-life workforce often comprises various professions of varying seniority and work experience. Teamwork is often advocated and practiced to varying degrees of success, depending on the leadership and team members' collaborative skills. While some might already acquire interprofessional collaborative skills from either previous training through work experience or both, others might not be practicing IPC at all, and the teams may merely delegate tasks among multi-professional teams without much interdependent work relationship. Thus, as fresh graduates join the real-life workforce, they encounter these different degrees of interprofessional collaborative practices. Knowing the concept of an actual interprofessional collaborative practice, fresh graduates might be puzzled over the difference between what they learned and encounter.

In Malaysia, medical and healthcare programs in universities have started to adopt the World Health Organization (WHO) IPCP Framework. The framework promoted IPL in the undergraduate curriculum. The curriculum is expected to prepare fresh graduates for IPCP in the health workforce (15–18). The IPL goal is to enable individual students to acquire knowledge, skills, and professional attitudes in ‘shared learning' through early contact with other professions (19, 20). The universities hope fresh graduates would be able to put into use various interprofessional skills they acquired during training. Given the challenges of the real-life workforce explained above, the transition from university interprofessional (IP) experience to work IP experience is worth examining to prepare students better for real-life IP work challenges. Therefore, this protocol focuses on how fresh graduate health professionals attempt to practice IPC when managing DM in a primary care setting and whether IPL experiences during undergraduate training influence the process of practicing IPC by these fresh graduate health professionals.

## 2. Methods

### 2.1. Research design

In health and social sciences, grounded theory (GT) is widely used to generate theoretical accounts of social phenomena which was developed by sociologists Glaser and Strauss (21). Unlike any other qualitative approaches, such as a case study or phenomenology where the bounded phenomena of interest or cultural understanding of a phenomenon are explored, GT methodology examines the processes of human actions and behavior from the data without predetermined concepts; the concerns of those involved are central to its understanding (21–24). This is in line with our research where we explore the processes and challenges of our primary care clinic fresh graduates health professionals who are managing DM on how they attempt to practice IPC at work. This method has its basis in symbolic interactionism where we assume individuals act on the meaning they assign to an object (e.g., social, attitude, behavior, belief, workplace, colleagues, and job demand) they interact with (25, 26).

### 2.2. Research team

This research comprises a dietitian (the first author) and three academic clinician-researchers with expertise in the interprofessional topic and research methodology. This research will be coordinated by the first author, a PhD candidate, with 11 years of academic working experience as a dietetics lecturer, who wants to know what is taught to students on interprofessional education and learning in the curriculum as it is translated into the real workforce. The second, third, and fourth authors are experienced academicians with backgrounds in medical and interprofessional education. Additionally, the third author has substantial qualitative research experience, including GT. Data will be analyzed and discussed by this research team (i.e., the authors).

### 2.3. Setting and participants

The research settings will involve public and university primary care clinics. These clinics provide services to people with chronic diseases such as diabetes mellitus, hypertension, and hyperlipidemia. Patients with such conditions will pay a visit to primary care clinics to obtain the first level of treatment rather than tertiary outpatient clinics in the hospitals. In Malaysia, the health professionals involved in diabetes care are primary care practitioners, dietitians, diabetes educators, and pharmacists (27). We intend to recruit junior medical officers, dietitians, nurses, diabetes educators, pharmacists, podiatrists, occupational therapists, and physiotherapists. The eligibility criteria for research participants are those who are Malaysians, who are working full time and have been in the workforce for a duration from 6 months to 5 years, who are managing and consulting diabetes care, who had not received any postgraduate degree, who are willing to have their voice and video recorded, and who have provided consent.

During the initial period, participants who fulfilled the relevant research objectives criteria will be recruited purposively. Theoretical sampling will be engaged where the data from a specific group of research participants are collected to further develop and refine the properties of the theory categories until no new theory emerges (28). Approximately 25 participants consisting of fresh graduate health professionals who manage DM care working in primary care clinics will be recruited for data collection for both in-depth interviews (IDI) and focus group discussions (FGD). The number of participants has been suggested by (23), a renowned grounded theorist, which we suppose may suffice for small-size studies to achieve theoretical saturation.

### 2.4. Data collection

The data collection method for this research will involve IDI, FGDs, and participant observation based on the consolidated criteria for reporting qualitative research (COREQ). In the IDI, an in-depth semi-structured interviewing method will be conducted on individual participants by the first author. Individual participants will be asked open-ended questions. The interview questions aim to stimulate participants to freely describe and explain their understanding of IPC, their experiences in IPL during their undergraduate studies, and the processes of practicing IPC in their work setting. Participants would be encouraged to “think aloud.” When necessary, participants will be probed during the interview without deviating from the research objective. Depending on participants' preferences and conveniences, a face-to-face or online web interview will be conducted. Permission to voice or video record will be obtained from the participants. The duration of an interview session will be approximately 45 minutes until an hour.

Two to three focus group meetings will be conducted as our second data source. A semi-structured interview guide will be created to structure the focus group meeting discussions. The interview guide consists of interview questions that will be constructed after the completion of IDIs. These questions will be further used as the extension of completed IDI data analysis findings. Through this method, we aimed to gain more in-depth knowledge and understanding of IPC and participants' practices from the perspective of a collective voice. Permission to voice or video record will be obtained, and the FGDs are expected to run for an hour to 1.5 hours. Similar to IDI, the focus group meetings will be conducted by either face-to-face or online web interviews, depending on participants' preferences and convenience. We will continuously recruit and conduct new rounds of interviews with new participants and previous participants while analyzing the data. We will also be flexible in our recruiting criteria based on what we learn but adhere to the research inclusion and exclusion criteria.

In the last data source, non-participatory observations would be conducted while the participants are at their workplace, carrying out their daily duty after the completion of FGDs data analysis. The purpose of observations is to immerse into the world to investigate and gain knowledge within the research project world (29). We will be able to experience the reality and discover further if there are any additional data for IDIs and FGDs (30). During observations, the phenomenon will be recorded, which participants might not be aware of or be unable to articulate the crucial details during IDIs and FGDs. Intermittent questions will be asked to explore the fundamental aspects of the research or any unclear situations or any situations which are inconsistent with what was reported in the interviews. The discreet annotation will be taken on participants' work settings, conversations, and actions as additional interaction information.

Interviews and discussions from IDIs and FGDs conducted in English will be transcribed verbatim using NVIVO 10 version. Those interviews in the Malay language will be translated and transcribed into the English language verbatim. Audio or video recordings of non-English interviews will be audited by another expert party to ensure the accuracy of the translated data and cultural understanding. All transcripts from all data sources will be checked against the recordings to ensure content accuracy.

### 2.5. Data analysis

Data collection and analysis will be conducted concurrently as part of the GT approach. There will be a repetitive process of data collection, coding of the transcripts, comparison, memoing, classification, and writing. Triangulation of data from IDIs, FGDs, and participant observations will be conducted. There will be three phases of coding, and constant comparison techniques will be applied throughout these coding processes. For the coding of the transcripts, we will continue to use the coding software NVIVO 10.

In the first phase, open coding will be conducted. This involves fracturing or breaking down the data into discrete parts and comparing them to develop information categories at a later stage (21, 23, 30). In this phase, we will select excerpts from the transcript through line-by-line reading and memos written on particularly interesting codes (23). Elements of the text that represent common concepts will be identified and then given a label or code (30).

In the second phase, axial coding will be conducted. We will identify the interrelationships between the concepts to create the main and subcategories of the codes generated from the axial coding phase by looking for recurring action about the “phenomenon,” “interaction,” and “group, individual, and collective” elements (30). To ensure our codes are compatible, the generated codes and data will be constantly compared during the coding process.

A discursive set of theoretical propositions will be formed in the final phase through theoretical coding and saturation. It will involve identifying a single-core category, and the theory will be consolidated (30). The theory will be discovered through this single-core category which can define and draw all the codes, sub, and main categories together.

Throughout the research, memos will be used to prompt and capture any reflections on the data. The memos will include operational processes relating to data collection and conceptual notes during the coding processes. The three data sources will be cross-checked to meet the credibility, appropriateness, accuracy, and confirmability criteria. Research rigor or trustworthiness would be ensured throughout the research with regular meetings with the research team (29). The research team will discuss and monitor the ongoing data collection and analysis, memo reflection, and resolve any discordance in the coding process.

### 2.6. Ethical consideration

Ethical approval has been obtained from the University Kebangsaan Malaysia Medical Research Ethics Board (JEP-2021-639) and the Ethics and Medical Research Committee, the Ministry of Health Malaysia [NMRR-21-1646-60768 (IIR)]. We will seek written consent from the participants to be interviewed, audio or video recorded, and observed before each IDI or FGD or participant observation. Each participant will be briefed on the purpose of the research, the data collection processes, and the expectations from them. Assurance will be given to participants that their identities are adequately protected, and the data will be kept strictly confidential. Participants are allowed to freely withdraw at any period during the research.

## 3. Discussion

### 3.1. Implication and usage

The IPCP involves more than one health professional from different professions working together with each other, patients, and families to coordinate and deliver comprehensive care plans (31). Practicing IPCP involves an interactive process of joining action among individuals (25, 26, 32, 33). Individuals are constantly engaged in “mindful action,” which constructs and negotiates the meaning of situations before an interaction happens. Furthermore, individuals' interactive behavior changes within a social environment based on the ongoing construction of these meanings. Social symbols are assigned based on these meanings. The social symbols could be the system, person, and things that arise from the constant interactions with one another. To investigate these interactions, the GT approach was selected because the GT approach focuses on the complex social processes such as social relationships and behaviors of groups through the construction of the meaning of the symbol (32). GT emphasizes the interaction between factors which can identify patterns of association between factors on the ground compared with abstract correlations through quantitative surveys since data will be obtained through the sufficient depth of actual individual's life actions and experiences (21). Furthermore, the GT approach allows the construction of a substantive framework based on the data rather than predefined past concepts. Past concepts and literature must earn their way to the framework as the substantive framework being constructed. The GT method has its limitations. Each coding stage in GT will be invariably time-consuming as it requires attention to all data and involves constant comparisons. Hence, the process is deemed slow. Furthermore, the GT method focuses more on an individual's action and less on examining the issues in an organization, which necessitates a case-study design. Nevertheless, it is our interest to focus on individual perspectives for the betterment of IPL design in undergraduate curriculum.

Technically, GT offers a systematic step to construct a framework explaining the process. From this research process, we hope to understand fresh graduates' challenges when translating what they have learned during their university studies or practicum experiences into day-to-day practice. Meanwhile, we hope that the findings from this research through the generated categories will form a framework showing the interrelationships of collaborative processes factors when fresh graduate primary care health professionals manage diabetes care in clinics. This framework will then function as a guideline and quality improvement to empower primary care fresh graduates to practice IPC successfully in their practice. We also hope that there are potential areas that emerged from the research findings to focus on while crafting interprofessional education and learning structure in medical and health sciences curricula that aimed to better prepare our fresh graduate health professionals for IPCP.

## Data availability statement

The original contributions presented in the research are included in the article, further inquiries can be directed to the corresponding author.

## Ethics statement

This research involving human participants were reviewed and approved by University Kebangsaan Malaysia Medical Research Ethics Board and the Ethics and Medical Research Committee, the Ministry of Health Malaysia. The participants will provide their written informed consent to participate in this research.

## Author contributions

KN, ST, and AA were responsible for the development and refinement of the protocol. KN came up with the idea for this project and wrote the draft and final manuscripts. ST provided substantial intellectual input into the methodology and provided an overall review of the structure. ST, AA, and MM contributed to the research topic and critical review, editing, and final approval of the manuscript version to be published. All authors contributed to the article and approved the submitted version.
